# Introducing flowable resin composite as a novel palatal dressing after free gingival graft harvesting: a randomized clinical trial

**DOI:** 10.1038/s41405-025-00362-4

**Published:** 2025-08-25

**Authors:** Mohamed Elsayed Temraz, Nesma Shemais, Eman Khalil, Dalia Ghalwash, Ahmed Elbarbary

**Affiliations:** 1 Oral Medicine and Periodontology Department, Faculty of Dentistry, The British University in Egypt, El- Sherouk City, Egypt; 2 Oral Medicine and Periodontology Department, Faculty of Dentistry, Cairo University, Cairo, Egypt; 3 Oral Medicine and Periodontology Department, Faculty of Dentistry, Galala University, Suez, Egypt

**Keywords:** Dentistry, Periodontics

## Abstract

**Objectives:**

This study aimed to evaluate the effectiveness of using a flowable resin composite compared to periodontal pack in reducing postoperative morbidity after free gingival graft (FGG) harvesting.

**Materials and methods:**

In this randomized controlled clinical trial, 34 patients requiring FGG were allocated into two equal groups. The intervention group received a flowable resin composite dressing over the palatal wound, while the control group received Coe-Pak. Postoperative pain was assessed using a Visual Analogue Scale (VAS) and mean analgesic consumption over 14 days. Secondary outcomes included wound size and color match of the healing site assessed at weeks 1, 2, 3, and 6.

**Results:**

The control group reported significantly higher VAS pain scores than the intervention group on days 1–4 and 10 (*p* < 0.05), and highly significant differences on days 5, 6, 8, and 9 (*p* < 0.001). Analgesic use decreased significantly in both groups (*p* < 0.001), with no intergroup differences. Wound size and color match improvements were comparable between groups.

**Conclusion:**

The application of flowable resin composite significantly reduces early postoperative pain following FGG harvesting without compromising healing outcomes. Its ease of application, patient comfort, and comparable clinical performance make it a promising alternative to traditional dressings. However, further investigations are warranted to confirm the biocompatibility of flowable resin composites and their impact on soft tissue healing.

## Introduction

A sufficient width of keratinized gingiva is essential for maintaining peri-implant and periodontal health, as confirmed by numerous clinical studies and systematic reviews [1–3]. Gingival grafting procedures are utilized to treat mucogingival deformities and gingival recession, aiming for root coverage and increasing keratinized tissue (KT) [4]. Soft tissue augmentation by autogenous grafts is the most predictable technique for maintaining peri-implant health by increasing tissue width and thickness [1, 5, 6].

FGG harvested from the hard palate, which is the standard donor site for autogenous graft harvesting [7, 8], is primarily indicated for increasing the width of keratinized tissue, especially in cases of mucogingival defects or a lack of attached gingiva. Unlike subepithelial connective tissue grafts, FGGs are not intended to augment soft tissue thickness, but rather to establish a stable zone of keratinized mucosa, which contributes to improved plaque control, peri-implant tissue health, and long-term periodontal stability [1, 2, 9]. An epithelialized gingival graft (EGG) may be applied directly to the recipient site or de-epithelialized to form a subepithelial connective tissue graft (CTG). This approach results in a graft that is denser, more stable, and less prone to shrinkage compared to conventional CTG harvesting techniques [10].

The FGG harvesting technique is advantageous for thin palatal mucosa, providing a faster, higher-quality graft with less fatty tissue and uniform thickness. It results in a denser, more stable graft with reduced shrinkage and can be used with or without the epithelium [10, 11].

Even though FGG is a reliable method for solving mucogingival issues like gingival recession, it is associated with higher donor site pain in the early postoperative phase [11–13].

The literature has documented several methods for protecting the donor site following the FGG procedure such as periodontal dressings, stents, and protective materials like Essix [14]. Furthermore, gelatin sponge with cyanoacrylate and hyaluronic acid proved to enhance palatal donor site protection [15]. Coe-Pak (GC America Inc., Alsip, IL, USA) is one of the most widely used periodontal packs and serves as the standard for evaluating new materials [16]. However, an ideal agent for this purpose is not highlighted in the pertinent literature [17].

Flowable resin composites were developed as restorative dental materials characterized by their low filler content and flowable features [18]. In periodontal plastic surgery, resin composites have been evaluated in clinical trials for treating combined defects, especially gingival recession associated with non-carious cervical lesions [19]. Moreover, flowable resin composite is often used to create customized temporary prostheses that shape soft tissues after implant placement [20].

Recently, attention has turned toward the use of flowable resin composite as a protective dressing over palatal donor sites. Meza-Mauricio et al. (2023) demonstrated, in a randomized controlled trial, that combining flowable composite with a collagen sponge significantly reduced postoperative pain and analgesic consumption compared to sponge alone [21]. Similarly, Belal (2025) reported favorable clinical outcomes in a case series using a suture-stabilized composite dressing, with high patient satisfaction and dressing stability throughout the healing period [22]. These findings highlight the potential of flowable composite as a minimally invasive, chairside solution for enhancing donor site management.

Accordingly, the purpose of the present study was to clinically compare the effects of directly applying flowable resin composite over the wound versus using Coe-Pak as a palatal wound dressing. The comparison focused on the incidence and severity of postoperative pain, the amount of analgesic consumption, and the healing of palatal wounds after free gingival graft harvesting.

## Materials and methods

### Study design

This was a randomized two-arm parallel clinical trial, performed in the Oral Medicine and Periodontology Department, Faculty of Dentistry, Cairo University, from March 2023 to December 2024.

The study was conducted in accordance with the ethical principles of the Declaration of Helsinki and adhered to the CONSORT guidelines for reporting randomized clinical trials. Ethical approval was granted by the Research Ethics Committee, Faculty of Dentistry, Cairo University (approval number: 8 | 12 | 22) and informed consent was obtained from all participants prior to their inclusion in the study. The research was pre-registered on www.clinicaltrials.gov. (registration number: NCT05779800).

### Sample size

Based on a prior study [23] reporting a standard deviation of 9 and a mean difference of 10 between experimental and control groups, a sample size of 14 participants per group was determined to be sufficient to achieve 80% power to detect a statistically significant difference. This calculation was conducted using a two-sided *t*-test assuming equal variances and a significance level of 0.05. This number was increased to 17 to account for potential dropouts during the follow-up period.

The sample size calculation was reviewed and approved by the Medical Biostatistics Unit (MBU) at the Faculty of Dentistry, Cairo University, prior to study initiation to ensure methodological rigor and appropriateness.

### Participants

#### Inclusion criteria: patients with mucogingival defects scheduled for FGG or CTG

Exclusion criteria: Smoking, presence of any uncontrolled local or systemic disease contraindicating periodontal plastic surgery, history of recent periodontal surgery at the donor site, severe gagging reflex, pregnancy and lactation, and patients allergic to the used agents.

#### Randomization and allocation concealment

Patients were randomly assigned (1:1) using computer-generated numbers (www.randomizer.org) to receive either flowable resin composite or Coe Pak for palatal wound dressing. Allocation was concealed and managed by a designated investigator (NS) based on the randomized sequence after donor site preparation.

### Blinding

The outcome assessor and biostatistician were blinded, whereas blinding was not feasible for participants and the clinician.

### Preoperative phase

All patients received nonsurgical periodontal therapy and oral hygiene instruction prior to grafting. Participants demonstrated stable periodontal conditions, and each underwent soft tissue grafting at a single site. While many grafts were used for treating gingival recession, others were placed for soft tissue augmentation in preparation for or around implants.

### Surgical procedure

The primary surgical site was prepared for soft tissue grafting, and graft dimensions were recorded. FGG was harvested following Zucchelli et al.‘s technique [24], involving two horizontal incisions, one 2 mm apical to the gingival margin, and two vertical incisions to outline the graft. The blade was initially inserted perpendicular to the bone, then redirected parallel to the palate to elevate a uniformly thick graft (1–1.5 mm) while preserving the palatal periosteum. The graft was immediately placed on sterile saline-soaked gauze to prevent shrinkage.

Most grafts were harvested by the same experienced periodontist (MT), with three cases in the control group and two in the intervention group performed by equally trained colleagues, all following a standardized technique and graft thickness of approximately 1–1.5 mm.

### Management of the palatal wound

**Fig. 1:**
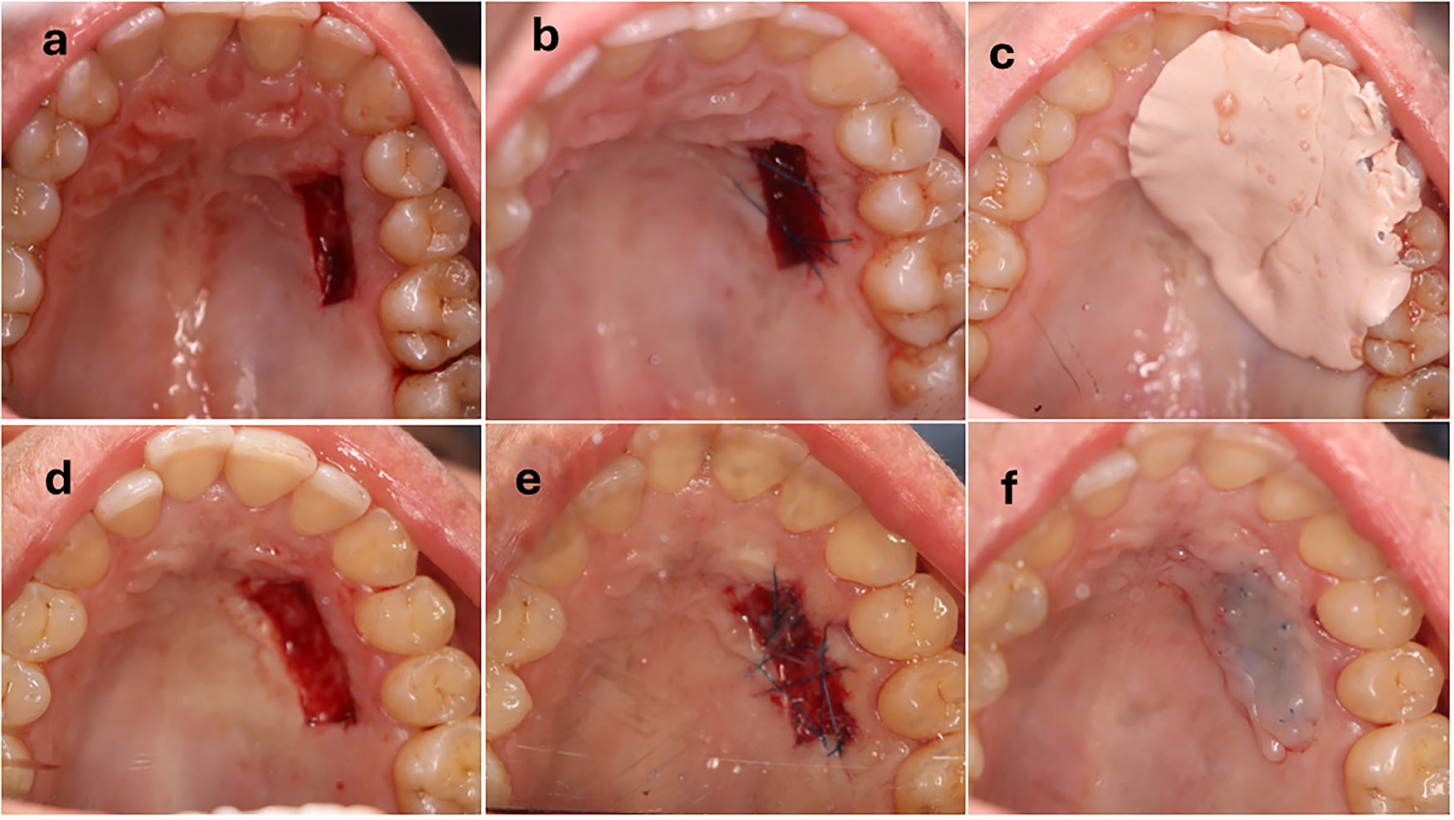
Clinical photographs of the palatal wound management. **a**–**c** Control group: palatal wound (**a**), after suturing (**b**), and after application of Coe-Pak dressing (**c**). **d**–**f** Intervention group: palatal wound (**d**), after suturing (**e**), and after application of flowable resin composite (**f**).

Following FGG harvesting, hemostasis was achieved by applying sterile gauze pressure for five minutes. The palatal wound was sutured using 5-0 polypropylene in a mesh-like pattern to retain the protective material. In the control group, the wound was covered with Coe-Pak (GC America Inc., Alsip, IL, USA), while in the intervention group, a continuous layer of flowable resin composite (Meta Biomed Co., Ltd., Chungcheongbuk-do, Korea) was applied and light-cured, mechanically interlocking with the sutures to secure it in place (Fig. 1).

### Postoperative care

Postoperative care included amoxicillin–clavulanic acid (1 g, twice daily for 6 days), and ibuprofen (600 mg on the day of surgery, with additional doses taken as needed and recorded to indirectly assess pain levels) for pain control [25]. Patients were instructed to rinse twice daily with 0.12% chlorhexidine for two weeks, avoid trauma to the surgical site for three weeks, and then begin gentle brushing using a soft toothbrush. All patients were instructed to follow a soft, non-irritating diet during the early postoperative period, including foods such as mashed vegetables, yogurt, eggs, and cooled soups. They were advised to avoid hot, hard, or spicy foods, acidic beverages, and alcohol. Patients were also encouraged to chew on the side opposite the donor site to minimize trauma during healing.

**Fig. 2:**
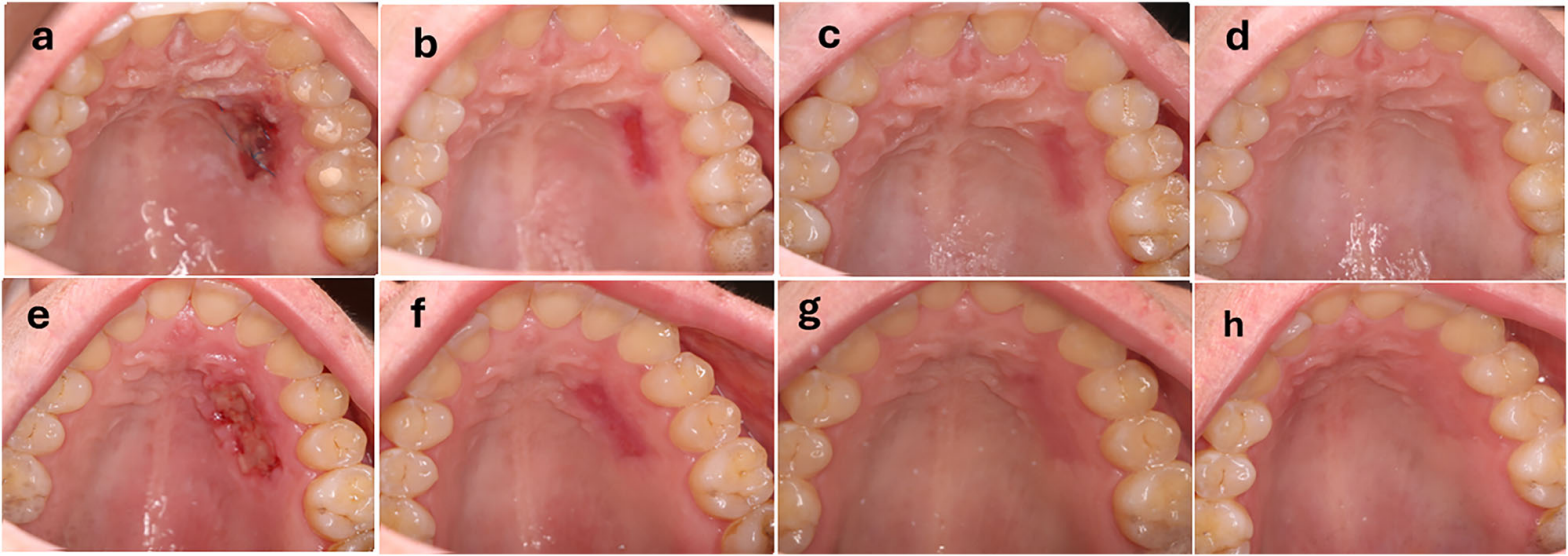
Clinical photographs illustrating palatal wound healing in both groups. **a**–**d** Healing progression in the control group at week 1 (**a**), week 2 (**b**), week 3 (**c**), and week 6 (**d**). **e**–**h** Healing progression in the intervention group at week 1 (**e**), week 2 (**f**), week 3 (**g**), and week 6 (**h**).

The palatal protective dressing was removed after one week in both groups [24]. Wound healing was monitored via clinical photographs taken at 1, 2, 3, and 6 weeks postoperatively (Fig. 2) [26].

### Outcomes

#### Primary outcome

Postoperative pain was assessed using a Visual Analogue Scale (VAS), with scores ranging from 0 (no pain) to 100 (severe pain) [8]. Patients self-reported their pain levels daily for two weeks (Wyrębek et al. 2018b).

Postoperative pain was also evaluated indirectly by measuring the mean analgesic consumption over 14 days, recorded in milligrams [24]. Patients were instructed to record the number of tablets taken each day. This record served as a secondary, objective measure of pain and was used alongside the VAS scores to evaluate postoperative discomfort.

This dual assessment approach combining subjective pain scores with objective analgesic intake enhanced the validity and clinical relevance of pain evaluation.

### Secondary outcomes

#### Wound size

**Fig. 3:**
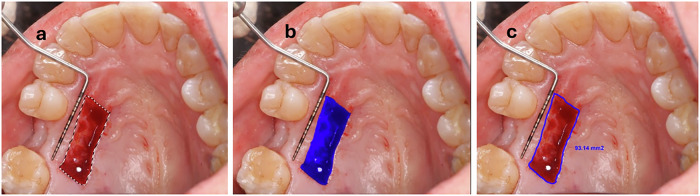
Clinical photographs illustrating the digital image analysis to assess the palatal wound area. Segmentation of wound boundaries using Adobe Photoshop 7.0 (**a**, **b**), and area quantification in mm^2^ using ImageJ software (**c**).

Wound size was measured intraoperatively with a UNC-15 periodontal probe [27]. Postoperative palatal wound size was accurately assessed using digital image analysis of photographs taken at baseline, and at weeks 1, 2, and 3. The analysis followed a two-step process: segmentation of wound boundaries in Adobe Photoshop 7.0 (Adobe Systems Inc., San Jose, California, USA), followed by area quantification in mm² using ImageJ software (version 1.53a, National Institutes of Health, USA) (Fig. 3) [28, 29].

#### Color match

Palatal mucosal color was objectively assessed at weeks 1, 2, 3, and 6 using a 0–10 VAS, comparing the graft site to adjacent tissues. Evaluation was performed by a blinded supervisor, with higher scores indicating better color match [14].

### Statistical analysis

Data normality was assessed using the Shapiro-Wilk test. Statistical analyses were conducted using SPSS software (version 25, IBM Corp., USA). Gender distribution between groups was compared using the Chi-square test, while age differences were analyzed using the independent t-test. Non-parametric data, including VAS pain scores, analgesic consumption, color match, and wound size, were analyzed using the Kruskal-Wallis test, followed by the Mann-Whitney U test for pairwise comparisons. A *p*-value ≤ 0.05 was considered statistically significant, while *p* ≤ 0.001 was regarded as highly statistically significant.

## Results

**Fig. 4:**
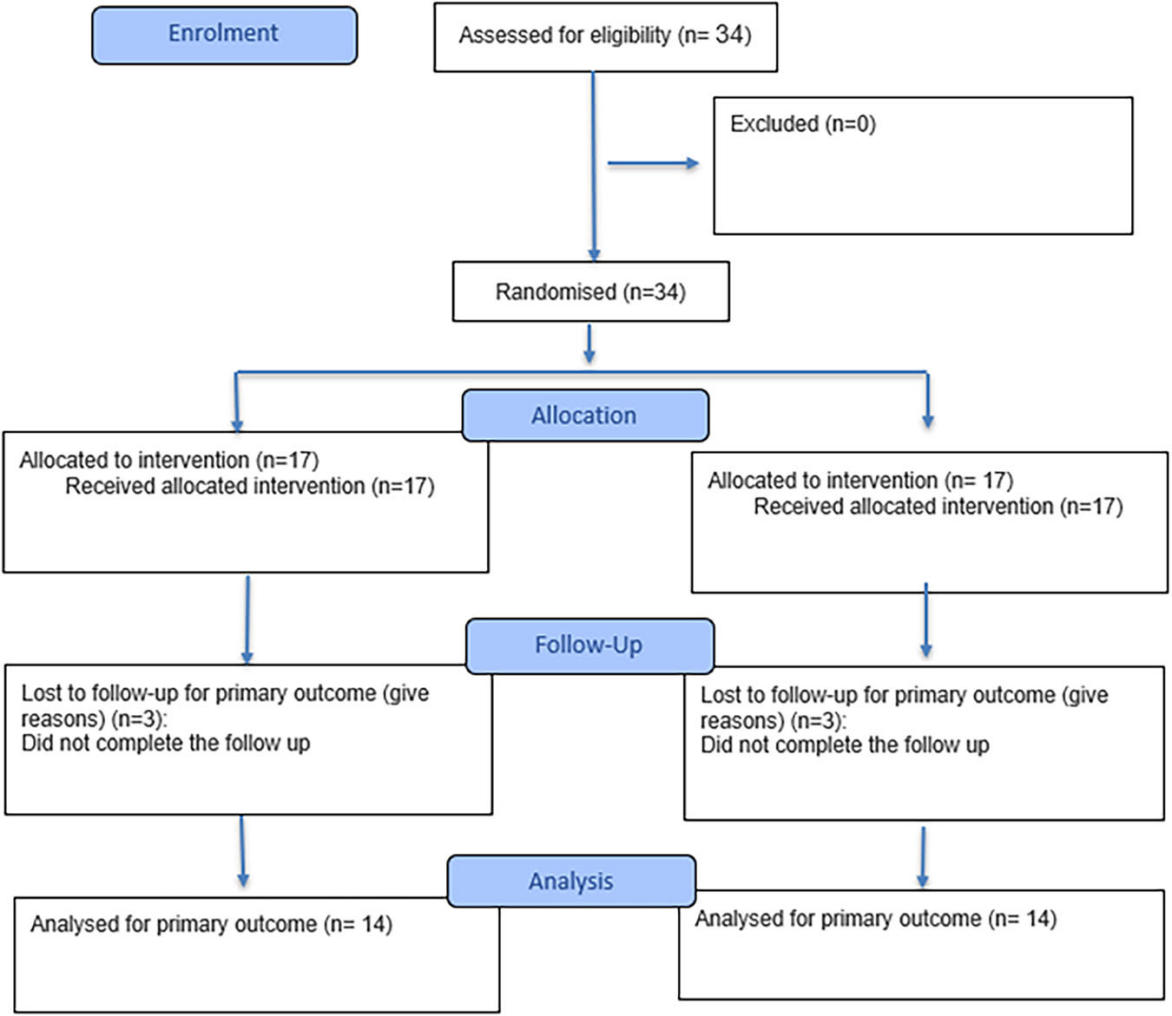
CONSORT flow chart of the randomized clinical trial.

A total of 34 patients were initially enrolled in this randomized controlled clinical trial, with 17 assigned to each group. Following the dropout of three participants in each group due to incomplete follow-up, 28 patients completed the study (Fig. 4). Chi-square analysis revealed no significant difference in gender distribution between groups (*p* = 0.893). The mean age was 38.71 ± 9.39 years in the intervention group and 38.71 ± 8.89 years in the control group, with no statistically significant difference detected (*p* = 0.989, independent t-test) (Table 1). During follow-up, dressing dislodgement occurred in eight cases in the control group (Coe-Pak) and in one case in the intervention group (flowable resin composite).

**Table 1:** Demographic data distribution and Inter-group comparison between the two studied groups.

		Control	Intervention	*P*-value^#^
**Gender**	**Male**	21.4% (3)	28.6% (4)	**0.893**^**NS**^
	**Female**	78.6% (11)	71.4% (10)	
**Age**		38.71 ± 9.39	38.71 ± 8.89	**0.989**^**NS**^

^#^*P*-value for Inter-group comparison between different materials calculated from (Chi-square test for Gender and Independent T-Test for Age). *NS* Non-significant *P* > 0.05.

### Assessment of pain score (VAS)

In both groups, postoperative pain scores progressively declined over 14 days. Control group initially presented a high median pain score (85), which decreased to zero by day 11, while intervention group started with a lower median score [28], reaching zero by day 5 with minor fluctuations. Kruskal-Wallis tests showed significant intra-group differences over time (*p* < 0.001 for control group; *p* < 0.05 for intervention group), reflecting a substantial reduction from peak to minimal pain by day 14 (Table 2).

**Table 2:** Basic Descriptive statistics of pain score (VAS) at different time intervals for both studied groups.

	Control		Intervention	
	Median	Range	Median	Range
**Day 1**	85	(0–100)	30	(0–100)
**Day 2**	65	(0–100)	20	(0–70)
**Day 3**	35	(0–90)	15	(0–70)
**Day 4**	25	(0–80)	10	(0–60)
**Day 5**	50	(0–100)	0	(0–60)
**Day 6**	50	(0–70)	0	(0–80)
**Day 7**	35	(0–100)	15	(0–90)
**Day 8**	40	(0–90)	0	(0–70)
**Day 9**	40	(0–100)	0	(0–70)
**Day 10**	20	(0–90)	0	(0–70)
**Day 11**	0	(0–50)	0	(0–50)
**Day 12**	0	(0–40)	0	(0–40)
**Day 13**	0	(0–40)	0	(0–50)
**Day 14**	0	(0–30)	0	(0–50)

**Fig. 5:**
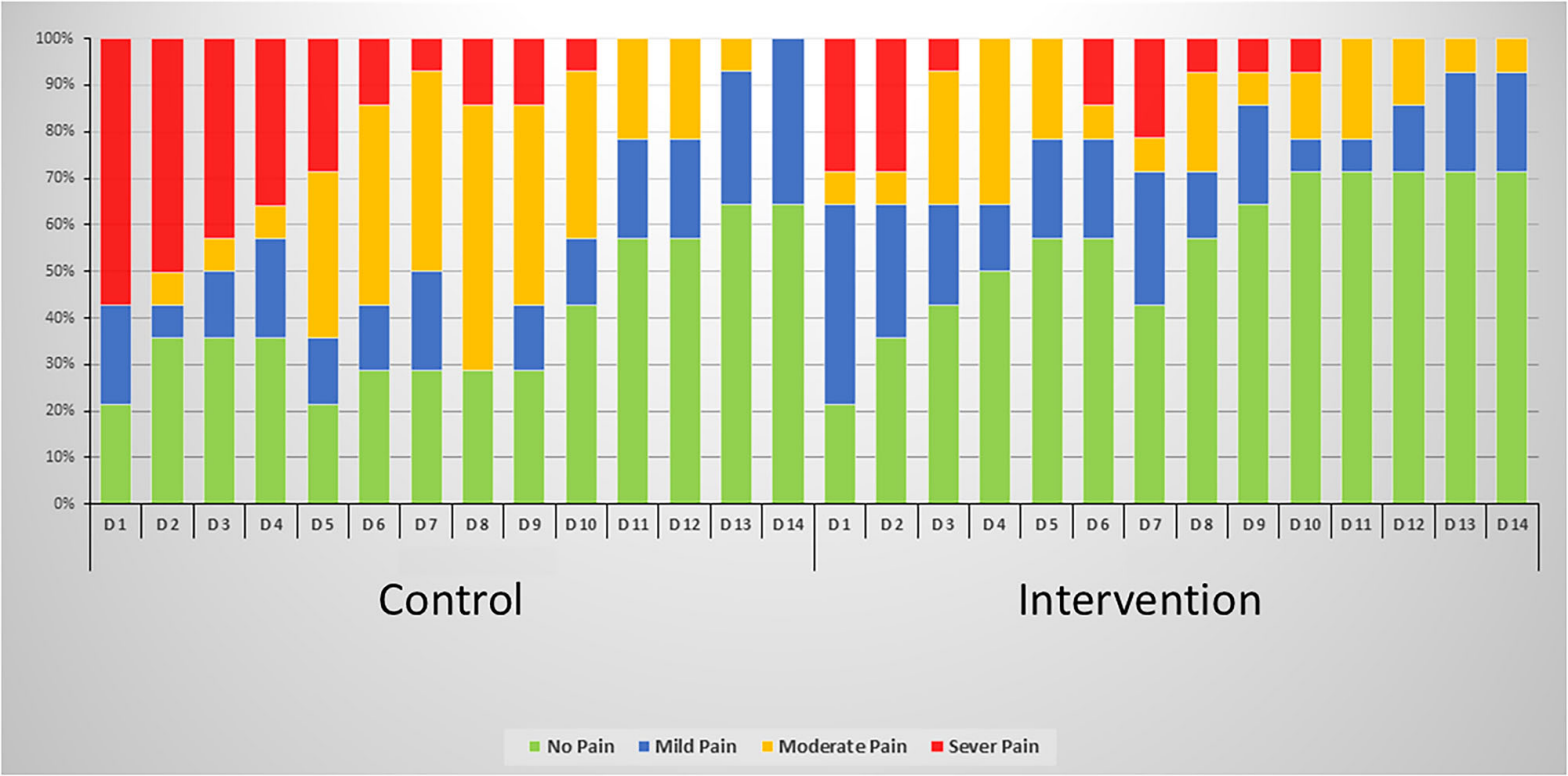
A bar chart demonstrates the distribution of VAS pain categories for both groups at different time intervals.

Intergroup comparisons (Table 3) revealed significantly higher pain scores in control group on days 1–4 and day 10 (*p* ≤ 0.05), and highly significant differences on days 5, 6, 8, and 9 (*p* ≤ 0.001). No significant differences were observed between groups from days 7 and 11–14, as pain levels approached zero in both groups. Additionally, the distribution of pain severity over time in both groups illustrated in (Fig. 5).

**Table 3:** Median, Range, intra, and inter-group comparison of pain (VAS) scores at different time intervals for both studied groups.

	Control		Intervention		*P-Value*^*#*^
	Median	Range	Median	Range	
**Day 1**	85^**A**^	(0–100)	30^**A**^	(0–100)	0.006*
**Day 2**	65^**B**^	(0–100)	20^**AB**^	(0–70)	0.007*
**Day 3**	35^**DE**^	(0–90)	15^**BC**^	(0–70)	0.010*
**Day 4**	25^**E**^	(0–80)	10^**C**^	(0–60)	0.018*
**Day 5**	50^**C**^	(0–100)	0^**D**^	(0–60)	<0.001**
**Day 6**	50^**C**^	(0–70)	0^**D**^	(0–80)	<0.001**
**Day 7**	35^**DE**^	(0–100)	15^**BC**^	(0–90)	0.058^**NS**^
**Day 8**	40^**CD**^	(0–90)	0^**D**^	(0–70)	<0.001**
**Day 9**	40^**CD**^	(0–100)	0^**D**^	(0–70)	<0.001**
**Day 10**	20^**E**^	(0–90)	0^**D**^	(0–70)	0.017*
**Day 11**	0^**F**^	(0–50)	0^**D**^	(0–50)	0.672^**NS**^
**Day 12**	0^**F**^	(0–40)	0^**D**^	(0–40)	0.869^**NS**^
**Day 13**	0^**F**^	(0–40)	0^**D**^	(0–50)	0.873^**NS**^
**Day 14**	0^**F**^	(0–30)	0^**D**^	(0–50)	0.937^**NS**^
***P-Value***^***##***^	<0.000**		0.027*		

^#^*P*-value for Inter-group comparison between the two groups (Mann-Whitney test).

^##^Overall *P*-value for Intra-group comparison between the different time intervals (Kruskal-Wallis test). Capital letters for intra-group comparison between the different time intervals (Mann-Whitney test) and the medians with different superscripts are statistically significant different at *P* ≤ 0.05.

**Highly significant at *P* ≤ 0.001, *Statistically significant at *P* ≤ 0.05.

*NS* Non-significant *P* > 0.05.

### Assessment of analgesics dosage

Analgesic use was consistent at a median of 1200 mg for the first three days, dropping to zero mg from day four in both groups. The Kruskal-Wallis test showed a significant decrease in analgesic use over time (*p* < 0.001), while the Mann-Whitney test found no significant differences between groups (*p* > 0.05) (Table 4).

**Table 4:** Median, Range, Intra, and Inter-group comparison of analgesics dosage (mg) at different time intervals for both studied groups.

	Control		Intervention		*P-Value*^*#*^
	Median	Range	Median	Range	
**Day 1**	1200^**A**^	(0–1800)	1200^**A**^	(0–1800)	0.435^**NS**^
**Day 2**	1200^**A**^	(0–1800)	1200^**A**^	(0–1800)	0.489^**NS**^
**Day 3**	1200^**A**^	(0–1800)	900^**A**^	(0–1800)	0.266^**NS**^
**Day 4**	0^**B**^	(0–1800)	0^**B**^	(0–1200)	0.523^**NS**^
**Day 5**	0^**B**^	(0–1800)	0^**B**^	(0–1200)	0.806^**NS**^
**Day 6**	0^**B**^	(0–1200)	0^**B**^	(0–1200)	0.907^**NS**^
**Day 7**	0^**B**^	(0–1200)	0^**B**^	(0–1200)	0.728^**NS**^
**Day 8**	0^**B**^	(0–1200)	0^**B**^	(0–600)	0.521^**NS**^
**Day 9**	0^**B**^	(0–1200)	0^**B**^	(0–600)	0.521^**NS**^
**Day 10**	0^**B**^	(0–1200)	0^**B**^	(0–600)	0.521^**NS**^
**Day 11**	0^**B**^	(0–1200)	0^**B**^	(0–600)	0.521^**NS**^
**Day 12**	0^**B**^	(0–1200)	0^**B**^	(0–0)	0150^**NS**^
**Day 13**	0^**B**^	(0–600)	0^**B**^	(0–0)	0.317^**NS**^
**Day 14**	0^**B**^	(0–600)	0^**B**^	(0–0)	0.317^**NS**^
***P-Value***^***##***^	<0.000**		<0.000**		

^#^*P*-value for Inter-group comparison between the two groups (Mann-Whitney test).

^##^Overall *P*-value for Intra-group comparison between the different time intervals (Kruskal-Wallis test). - Capital letters for intra-group comparison between the different time intervals (Mann-Whitney test) and the medians with different superscripts are statistically significant different at *P* ≤ 0.05.

**Highly significant at *P* ≤ 0.001. *Statistically significant at *P* ≤ 0.05.

*NS* Non-significant *P* > 0.05.

### Secondary outcomes

#### Assessment of wound size

Both groups exhibited significant reductions in wound size over time. The most pronounced decrease occurred from day 0 to day 21. The Kruskal-Wallis test confirmed highly significant intra-group differences (*p* < 0.001), highlighting the substantial wound size reduction within each group. However, intergroup comparisons at each time point revealed no statistically significant differences between the groups (Table 5).

**Table 5:** Median, Range, Intra, and Inter-group comparison of wound size at different time intervals for both studied groups.

	Control		Intervention		*P-Value*^*#*^
	Median	Range	Median	Range	
**Baseline**	96.56^**A**^	(48.99–230.93)	111.58^**A**^	(67.88–164.99)	0.491^**NS**^
**Week 1**	70.13^**B**^	(36.01–158.48)	94.33^**A**^	(42.52–116.27)	0.198^**NS**^
**Week 2**	45.75^**BC**^	(12.72–148.25)	37.42^**B**^	(16.88–82.64)	0.408^**NS**^
**Week 3**	17.58^**C**^	(6.17–84.48)	14.19^**B**^	(4.39–44.91)	0.198^**NS**^
***P-Value***^***##***^	< 0.000**		< 0.000**		

^#^*P*-value for Inter-group comparison between the two groups (Mann-Whitney test).

^##^Overall *P*-value for Intra-group comparison between the different time intervals (Kruskal-Wallis test). Capital letters for intra-group comparison between the different time intervals (Mann-Whitney test) and the medians with different superscripts are statistically significant different at *P* ≤ 0.05.

**Highly significant at *P* ≤ 0.001, *Statistically significant at *P* ≤ 0.05.

*NS* Non-significant *P* > 0.05.

#### Assessment of the color of the palatal mucosa

Both groups demonstrated significant improvements in palatal mucosa color match over time, as detailed in (Tables 6 and 7). The Kruskal-Wallis test showed a highly significant temporal effect within each group (*p* < 0.001), while the Mann-Whitney test found no significant differences in color scores between groups at any time point (*p* > 0.05).

**Table 6:** Basic Descriptive statistics of color of the palatal mucosa at different time intervals for both studied groups.

	Control		Intervention	
	Median	Range	Median	Range
**Week 1**	4	(2–6)	3	(2–5)
**Week 2**	5	(3–8)	6	(5–7)
**Week 3**	7	(5–8)	8	(6–8)
**Week 6**	9	(8–10)	9	(8–10)

**Table 7:** Median, Range, Intra, and Inter-group comparison of color matching at different time intervals for both studied groups.

	Control		Intervention		*P-Value*^*#*^
	Median	Range	Median	Range	
**Week 1**	4^**C**^	(2-6)	3^**C**^	(2-5)	0.234^**NS**^
**Week 2**	5^**BC**^	(3-8)	6^**B**^	(5-7)	0.231^**NS**^
**Week 3**	7^**AB**^	(5-8)	8^**AB**^	(6-8)	0.135^**NS**^
**Week 6**	9^**A**^	(8-10)	9^**A**^	(8-10)	1.000^**NS**^
***P-Value***^***##***^	<0.000**		<0.000**		

^#^*P*-value for Inter-group comparison between the two groups (Mann-Whitney test).

^##^Overall *P*-value for Intra-group comparison between the different time intervals (Kruskal-Wallis test). - Capital letters for intra-group comparison between the different time intervals (Mann-Whitney test) and the medians with different superscripts are statistically significant different at *P* ≤ 0.05.

**Highly significant at *P* ≤ 0.001, *Statistically significant at *P* ≤ 0.05.

*NS* Non-significant *P* > 0.05.

## Discussion

FGG remains the best choice, particularly for increasing the width of the keratinized mucosa [30] as it is a relatively fast procedure, provides a higher quality graft with less glandular and fatty tissue and more uniform thickness [24]. However, pain in the donor site has been reported in various studies as the most frequent postoperative complication following FGG or CTG procedures [13, 31].

Several clinical trials have attempted to reduce postoperative complications in patients following soft tissue graft harvesting utilizing various materials such as absorbable collagen sponge [32], Alvogyl [33], cyanoacrylate [31], and acrylic stents and propylene mesh, which yielded positive outcomes [34]. In addition to Coe-Pak, which is one of the most popular dressings on the market today, it provides a standard for new dressings to be measured against. However, it is not aesthetically acceptable to most patients [16].

This study evaluated the efficacy of a flowable resin composite stent compared to Coe-Pak in reducing postoperative pain and enhancing wound healing following FGG harvesting. The findings demonstrated that the flowable resin composite significantly reduced pain levels, particularly during the early postoperative phase, as evidenced by lower VAS scores. Statistically significant differences were observed on the first four days and day 10 (*p* < 0.05), with highly significant differences on days 5, 6, 8, and 9 (*p* < 0.001). These results suggest that the superior pain control provided by the resin composite may be attributed to its stable and protective coverage of the wound during the initial healing phase, in contrast to the less stable Coe-Pak dressing, which may disintegrate, act as an irritant to the palatal wound, or become dislodged prior to its scheduled removal.

Regarding pain category distribution, most patients in the control group experienced severe pain during the first 4 days. On the other hand, most patients in the intervention group experienced mild pain.

These findings agree with a recent clinical study that evaluated the effect of using flowable resin composite for pain management after FGG harvesting and concluded that the addition of flowable resin composite coating to the hemostatic collagen sponge on the palatal wound helped to minimize postoperative pain [21].

Similarly, Belal (2025) reported favorable outcomes in a case series where flowable resin composite was applied in combination with a suture-stabilized Alvogyl dressing for palatal wound coverage. The study demonstrated reduced postoperative discomfort, improved dressing retention, and high patient satisfaction. Although the dressing combination differed from our protocol, the clinical advantages observed further support the potential of flowable resin composite as a protective and patient-friendly palatal dressing [22].

The reduction in analgesic consumption was statistically significant in both groups (*p* < 0.001), reflecting the marked reduction in medication requirements between the initial assessment at day 1 (highest dosage) and the final assessment at day 14 (lowest dosage). These findings are consistent with prior studies demonstrating the benefits of protective barriers in minimizing postoperative discomfort and enhancing healing in soft tissue grafting procedures [34].

The current novel approach of directly applying the flowable composite on the palatal wound is easier, more cost-effective, and more timesaving because it eliminates the need to bond the flowable composite to the palatal surface of the teeth. Instead, we directly apply the flowable resin composite to the palatal wound after suturing. Moreover, our technique offers greater convenience when grafts are harvested from a palatal site near an edentulous area where no teeth are available for bonding. Additionally, this approach eliminates the need for etching and bonding to healthy tooth structure.

The present results revealed comparable wound-healing efficacy of the flowable composite with Coe-Pak, with both demonstrated similar wound size reduction by week three. This aligns with clinical findings indicating that the use of flowable composite in the fabrication of customized healing abutments in direct contact with soft tissue, after implant exposure, reduces bacterial contamination in the bone regeneration zone, limits soft tissue ischemia, and promotes both mucogingival and osseointegration processes. Additionally, it facilitates the rapid development of the desired emergence profile for the final prosthesis [20].

Like wound size, color match analysis at week six showed no significant difference between groups; however, both groups showed a highly significant difference in color match among time intervals in intragroup comparison. This significance indicates that both materials provide acceptable aesthetic and healing outcomes in the long term.

These findings have direct clinical implications for practitioners performing FGG procedures. The flowable composite appears to offer a superior pain management strategy in the immediate postoperative period, reducing patient discomfort and minimizing the need for analgesics. Given the comparable long-term healing outcomes of both techniques, clinicians may select either approach based on patient preference, material availability, ease of application, and final clinical results. These aspects particularly emphasize the advantages of using flowable resin composite.

This study had several limitations. Firstly, the relatively small sample size may have limited the generalizability of the findings. Secondly, the reliance on patient-reported pain assessments introduced inherent subjectivity, potentially affecting result consistency. Thirdly, while postoperative depth was not measured to preserve healing, graft thickness was standardized, and future studies should consider volumetric assessment for more comprehensive evaluation. Fourthly, minor operator variability occurred in five cases, despite adherence to a standardized grafting protocol. Lastly, although the dressing application protocol was standardized, visual inspection of photographs suggests minor variations in the contour or thickness of the applied materials, which may have influenced tissue response and pain perception.

Future research should involve larger sample sizes and employ objective pain assessment methods to enhance the validity of the outcomes. While no adverse tissue reactions were observed in the present study, the potential biological effects of flowable resin composite on wound healing remain an important consideration. Previous studies suggest the material is generally biocompatible; however, its impact on epithelialization, angiogenesis, and the local inflammatory response warrants further investigation. Future research should incorporate histologic or volumetric assessments to more comprehensively evaluate the short- and long-term effects of resin-based dressings on soft tissue healing.

## Conclusion

The application of flowable resin composite on palatal wounds following FGG harvesting appears to significantly reduce postoperative pain. Owing to its ease of use, availability, and favorable patient acceptance, this material demonstrates promising potential in enhancing postoperative comfort and wound management. However, further investigations are warranted to confirm the biocompatibility of flowable resin composites and their impact on soft tissue healing.

## Data Availability

The data that support the findings of this study are available from the corresponding author upon reasonable request.
